# Research Lumbar Puncture Recruitment, Safety and SOPs in Black/African American and white middle‐age participants

**DOI:** 10.1002/alz.090789

**Published:** 2025-01-09

**Authors:** Kelly D. S. Likos, William T. Hu, Danielle D Verble, Lynn Marie Trotti, Karima Benameur, Laura M Scorr, Brittany Butts, Whitney Wharton

**Affiliations:** ^1^ Emory University, Atlanta, GA USA; ^2^ Rutgers Institute for Health, Health Care Policy, and Aging Research, New Brunswick, NJ USA; ^3^ Emory University School of Medicine, Atlanta, GA USA; ^4^ Emory University, Altanta, GA USA

## Abstract

**Background:**

The Wharton Lab has collected CSF for over 10 years as a primary endpoint in NIH/NIA funded longitudinal observational studies and clinical trials in cognitively normal individuals with a parental history of Alzheimer’s disease (AD). LPs provide vital data on AD risk and progression and time specific information on efficacy for clinical interventions, yet few investigators include research LPs, for fear of clinical implications, lack of specialized clinicians, and perceived unwillingness of participants to consent to LPs, particularly in minoritized participants

**Method:**

Comprehensive data is collected on willingness to take part in LP, safety, and procedure specifics, including side effects. Twenty milliliters of CSF are obtained using fluid drip or aspiration methods. Twenty‐four hours post LP, the study team contacts the participant for LP follow up using a standardized questionnaire (potential side effects, severity, and home care initiated). Daily follow‐up with the participant occurs until side effects subside

**Result:**

Our data in 122 participants enrolled (1R01AG066203‐02) indicate that 1) show that research LPs are safe and well tolerated and 2) cognitively normal, middle‐aged participants are willing to undergo multiple LPs, regardless of race, ethnicity, and gender.

Participants were 51.1% Black or African American and 45.2% Non‐Hispanic White, 76.3% female and have an average BMI of 27.8. 9.6% of individuals self‐excluded because of LP requirement. Of 139 LPs preformed, 93.9% of LPs were successful. 13.1% of participants reported a headache 24‐hours post LP. 78.8% of participants reported no side effects. Headache(n=10) and lower back pain(n=4) were the most common side effects. When side effects occurred post LP, 16.3% initiated home care suggested by the research team, with hydration being the most common(n=6).

LPs were most successful with a 22 Whitacre needle, followed by a 22 Quincke needle

**Conclusion:**

Data show that research LPs are safe and well tolerated in diverse cohorts, and that individuals are willing to take part in research LPs. Regular LP discussion and education for potential subjects is encouraged. Increased education and discussion of research LPs with the research team provides opportunities to discuss the procedure with the potential participant during the recruitment, consent, and screening processes

